# Lasting Effects of a 2-Year Diabetes Self-Management Support Intervention: Outcomes at 1-Year Follow-Up

**DOI:** 10.5888/pcd9.110313

**Published:** 2012-06-07

**Authors:** Tricia S. Tang, Martha M. Funnell, Mary Oh

**Affiliations:** Author Affiliations: Martha M. Funnell, Mary Oh, University of Michigan Department of Medical Education, University of Michigan Medical School, and Michigan Diabetes Research and Training Center, Ann Arbor, Michigan.

## Abstract

**Introduction:**

Diabetes-related health improvements achieved from self-management education interventions are not sustained long-term. We examined the health effects at 1 year follow-up of a 2-year, empowerment-based, diabetes self-management support intervention designed for African Americans.

**Methods:**

We collected data from 52 African American adults with type 2 diabetes who completed the 3-year study. The intervention consisted of weekly groups led by 2 health care professionals and emphasized experiential learning, emotional coping, problem solving, goal setting, and action planning; group discussion was guided by participant-identified self-management priorities and concerns. Measurements were taken at baseline, 24 months (postintervention), and 36 months (1 year follow-up) to assess glycemic control; weight; body mass index; serum cholesterol, high-density lipoprotein cholesterol, and low-density lipoprotein cholesterol levels; systolic and diastolic blood pressure; self-care behaviors; diabetes-specific quality of life; and diabetes empowerment.

**Results:**

Following the 2-year diabetes self-management support intervention, we found significant improvements for following a healthy diet (*P* = .03), spacing carbohydrates evenly across the day (*P* = .005), using insulin as recommended (*P* = .047), and achieving diabetes-specific quality of life (*P* = .02). At 1-year follow-up, not only did participants sustain the behavioral improvements made in the 2-year diabetes self-management support intervention, but they also demonstrated additional improvements in glycemic control (*P* < .001) and in serum cholesterol (*P* < .001) and low-density lipoprotein cholesterol levels (*P* = .001).

**Conclusion:**

Participation in an empowerment-based diabetes self-management support intervention may have a positive and enduring effect on self-care behaviors and on metabolic and cardiovascular health.

## Introduction

Although considerable evidence supports the efficacy of diabetes self-management interventions in improving clinical, behavioral, and psychosocial outcomes (1-8), a 2001 systematic review conducted by Norris and colleagues (1) concluded that improvements achieved from self-management programs were short-lived, lasting no more than 6 months postintervention. However, of the 54 studies that examined glycemic control as an end point in this review, only 33% (n = 18) included a follow-up assessment at 12 months or longer, which means that most studies did not evaluate long-term outcomes. Since that review was published, a small but growing number of studies have investigated the long-term effects of self-management interventions (9-15).

The need to place more emphasis on lifelong self-management efforts has been reinforced by the National Standards for Diabetes Self-Management Education (DSME), which require that patients receive ongoing diabetes self-management support (DSMS) (16). Although the importance of ongoing DSMS is formally recognized, how much or what type of support is needed is unclear. Research examining long-term outcomes has used different models for supporting and sustaining self-management efforts including clinic-based group visits (9-11); intensive, structured hospital-based education (12); the chronic care model (13); Internet-based approaches (14); and combined education and skills-development training (15). Studies provide preliminary evidence that both simple and complex diabetes self-management interventions have the potential to yield enduring diabetes-related health benefits. Short-term improvements in glycemic control and quality of life achieved among African American women (N = 109) with type 2 diabetes following an 11-week coping skills and a 10-week conventional diabetes education intervention were sustained at 24 months (15). Similarly, health-related improvements (hemoglobin A1c [HbA1c], blood pressure, blood glucose monitoring) achieved from a 12-month diabetes intervention based on a chronic care model, compared with a usual care condition, persisted at 3-year follow-up (13).

As a strategy to foster long-lasting improvements, some interventions have built-in periodic reinforcement strategies during the follow-up period (9-12,14). In a study that compared group-based self-management support with individual self-management, quality of life improved for participants in the group-based self-management intervention, which repeated its 4-session curriculum (ie, provided reinforcement) each year for 5 years following the year-long intervention, while it deteriorated for participants in the individual self-management support control group. Furthermore, glycemic control remained steady for the intervention group but worsened for the control group (11). Findings suggest that self-management interventions incorporating reinforcement can help sustain improvements made in the short term as well as promote continuing improvement over a longer period.

Support is growing for the efficacy of DSME interventions in producing lasting diabetes-related health benefits. Existing literature has focused on assessing the long-term follow-up of short-term DSME interventions. To date, less attention has been paid to investigating the enduring effects of long-term DSMS interventions. The objective of this study was to examine the effect on clinical, self-care, and psychosocial outcomes of a 2-year empowerment-based DSMS intervention at 1-year follow-up.

## Methods

The University of Michigan institutional review board approved this study; methods have been described elsewhere (17,18). This investigation was part of a larger, single cohort, longitudinal, prospective study that was conducted in 3 phases. Phase 1 involved a 6-month mailed DSME intervention with clinical feedback. Phase 2 involved a 2-year ongoing DSMS intervention called the Lifelong Diabetes Self-Management Intervention (LMI). Phase 3 involved a post-DSMS intervention follow-up period in which participants received usual care and no intervention. This article focuses specifically on phases 2 and 3 of the larger study.

### The Lifelong Diabetes Self-Management Intervention

The LMI was designed on the basis of patient empowerment principles, which view diabetes as a patient-managed disease, emphasize the patient (vs provider) as the ultimate decision maker, and recognize that patient-selected (vs provider-selected) goals have a greater likelihood of being achieved and sustained (19). The intervention consisted of 88 weekly sessions, each lasting approximately 75 minutes, conducted over 24 months. We offered sessions at a local community center and at different times (10:00 AM and 3:00 PM) to accommodate participants’ schedules. We invited participants to attend sessions as frequently as they needed or were able, given their different support needs and competing life demands. Participants’ self-management questions and concerns guided group discussions. During each group session, facilitators encouraged participants to share self-management challenges, discuss emotions associated with those challenges, participate in group-based problem solving, raise questions about diabetes and its care, and set self-management goals and develop action plans to accomplish those goals. Sessions were conducted by a nurse certified diabetes educator and a clinical psychologist.

A detailed description of participant recruitment is published elsewhere (17). Briefly, we recruited African American adults with type 2 diabetes living in the greater Ypsilanti, Michigan, area, using various approaches (eg, flyers posted at local health and community centers, newspaper advertisements, invited presentations at local African American churches). People interested in participating called a free study hotline to be screened for eligibility. Criteria for inclusion were self-identifying as African American, being aged 40 or older, being diagnosed with type 2 diabetes for at least 1 year, having received some form of DSME in the past, and being under the care of a health care provider. Eligible people enrolled in the study and provided informed consent.

### Data collection

Baseline assessment was completed immediately before conducting the LMI (at the end of phase 1). We also conducted assessments at 24 months (at the end of phase 2) and at 36 months (at the end of phase 3). All assessments included a blood draw (HbA1c and lipid panel), weight and height measurement, blood pressure reading, and a self-report survey. To compensate participants for time and effort, we provided a $50 stipend on completion of each assessment.

### Measures

We measured clinical outcomes at face-to-face, group-based assessment sessions. We obtained HbA1c and a lipid panel (serum, high-density lipoprotein [HDL], and low-density lipoprotein [LDL] cholesterol) via venous puncture; measured systolic blood pressure (SBP) and diastolic blood pressure (DBP) using an appropriately sized automatic blood pressure cuff (Omron HEM780, Omron Healthcare Inc, Lake Forest, Illinois); and measured weight using the Health o meter Pro Series Spring scale (Jarden Consumer Solutions, Boca Raton, Florida) and height using a Seca 214 stadiometer (Seca gmbh and Co, Hanover, Maryland). We used weight and height measurements to calculate body mass index (BMI, in kg/m^2^).

The *Summary of Diabetes Self-Care Activities Measure, Revised* was used to assess self-management behaviors (20). We selected items including frequency (ie, during the past week; range, 0-7 days) of following a healthy diet; spacing consumption of carbohydrates evenly during the day; participating in physical activity; monitoring blood glucose levels; inspecting feet; and taking medication or insulin or both. Greater number of days was indicative of better self-management.

The Diabetes Distress Scale, a 17-item instrument that measures emotional distress and functioning as it relates to living with diabetes, was used to assess diabetes-specific quality of life (21). Responses are scored on a 6-point Likert scale (1 = no problem to 6 = serious problem). Score range for the Diabetes Distress Scale is 17 to 102; lower scores indicate a higher diabetes-specific quality of life.

Diabetes empowerment was assessed using the short form of the Diabetes Empowerment Scale, an 8-item scale assessing perceived ability to manage the psychological and social demands and challenges associated with diabetes (22). Items were scored on a 5-point Likert scale; higher mean scores indicate greater level of confidence managing one’s diabetes.

Demographic characteristics included age, sex, employment status, marital status, education level, annual household income, insurance coverage, years since diagnosis, and type of diabetes treatment.

At the start of the intervention, we recruited 77 participants, of which 52 (67%) were retained at the end of the 36-month study. The actual attrition rate (33%) was below the expected rate of 48% over the course of the 3-year study (20% per study year).

### Statistical analysis

We used descriptive statistics to assess attendance patterns and the demographic and diabetes care–related characteristics of the sample. We conducted independent sample *t* tests and Pearson χ^2^ or Fisher’s exact test (the latter for 2 × 2 distributions) to compare the demographic and clinical characteristics of participants who completed the study (“participants”) and participants who dropped out of the study before completion (“dropouts”). We performed paired *t* tests to examine pre–post changes associated with the 2-year LMI and 1-year follow-up periods. Finally, we used Pearson correlations to examine the relationship between frequency of attendance and health-related improvements associated with the 2-year intervention.

## Results

### Characteristics of the sample

Mean age of participants (N = 52) was 63.1 years (standard deviation, 10.3 y; range, 40-84 y); 73% of participants were female and 46% were currently married (Table 1). Thirty-three percent had a high school degree or less, 75% were not currently employed, and 52% had Medicare. No significant differences were found for either clinical or demographic variables between participants who completed the 3-year study and participants who dropped out (Table 1).

**Table 1:** Baseline Demographic and Clinical Characteristics for Participants (n = 52) and Dropouts (n = 25) of a 2-Year Diabetes Self-Management Support Intervention for African American Adults With Type 2 Diabetes, Ypsilanti, Michigan, 2006-2009^a^

Continuous Variables	Participants, Mean (SD)	Dropouts,^b^ Mean (SD)	*P* Value^c^
Age, y	63.1 (10.3)	58.3 (10.0)	.06
Years since diagnosis	11.8 (11.7)	12.9 (9.0)	.68
HbA1c, %	7.9 (1.7)	9.0 (2.8)	.08
Systolic blood pressure, mm Hg	137.9 (18.2)	136.9 (15.5)	.81
Diastolic blood pressure, mm Hg	74.9 (10.3)	78.1 (12.5)	.24
Serum cholesterol, mg/dL	153.1 (34.0)	146.9 (38.7)	.48
HDL cholesterol, mg/dL	52.8 (12.9)	51.7 (14.9)	.75
LDL cholesterol, mg/dL	96.2 (31.5)	88.8 (31.1)	.33

**Categorical Variables**	**Participants, n (%)**	**Dropouts,^b^ n (%)**	***P* Value^c^**

**Sex**			
Female	38 (73)	15 (60)	.30
Male	14 (27)	10 (40)	
**Marital status**			
Currently married	24 (46)	9 (36)	.77
Separated/divorced	15 (29)	7 (28)	
Widowed	6 (12)	4 (16)	
Other	7 (13)	5 (20)	
**Education level**			
8 grades or less	3 (6)	0	.36
Some high school	2 (4)	3 (12)	
High school graduate or GED	12 (23)	6 (24)	
Some college or technical school	25 (48)	9 (36)	
College graduate or higher	10 (19)	7 (28)	
**Annual household income, $**			
0-9,999	6 (12)	8 (33)	.17
10,000-19,999	13 (25)	5 (21)	
20,000-29,000	8 (15)	5 (21)	
30,000-59,000	13 (25)	3 (12)	
≥60,000	11 (22)	3 (12)	
**Employment status**			
Currently not working	13 (25)	4 (16)	.56
Not currently working	39 (75)	21 (84)	
**Insurance coverage**			
Individual plan	2 (4)	0	.06
Employee plan	19 (37)	6 (24)	
US governmental health plan	0	1 (4)	
Medicare	27 (52)	11 (44)	
Medicaid	4 (8)	7 (28)	
No health insurance	0	0	
**Diabetes treatment^d^**			
Using insulin	14 (27)	12 (48)	.12
Taking pills	41 (79)	27 (75)	.57
Using Byetta	2 (4)	1 (3)	>.99
Using Symlin	0	0	>.99
Taking cholesterol pills	41 (80)	15 (60)	.09
Taking blood pressure pills	48 (92)	20 (80)	.14

Abbreviations: SD, standard deviation; HbA1c, hemoglobin A1c; HDL, high-density lipoprotein; LDL, low-density lipoprotein; GED, general educational development; NA, not applicable.

^a^ Values may not sum to n because of missing data.

^b^ Dropouts were defined as participants who did not complete the 1-year follow-up assessment.

^c^ We conducted independent sample *t* tests and Pearson χ^2^ or Fisher exact tests (the latter for 2 × 2 distributions) to compare the demographic and clinical characteristics between participants and dropouts. None of the comparisons reached significance.

^d^ More than 1 option could be chosen for this category.

### Attendance patterns

Of total participants, 58% (n = 30) attended 3 to 23 sessions, 21% attended 24 to 44 sessions, and 21% attended 45 to 83 sessions. Mean attendance for morning sessions was 11; mean attendance for afternoon sessions was 4. On average, 29% of the total sample attended each week.

### 2-Year intervention period

Table 2 presents participants’ clinical, self-care, and psychosocial measures before and immediately following the 2-year LMI (ie, phase 2 of the study). No significant changes were found for any clinical outcomes. For self-care behaviors, significant improvements were found for following a healthy diet (*P* = .03), spacing carbohydrates evenly throughout the day (*P* = .005) and insulin use (*P* = .047). No changes were found for physical activity, blood glucose testing, foot care, or medication use. A significant improvement was found for diabetes-specific quality of life (*P* = .02). No changes were found for the diabetes empowerment measure. Frequency of attendance was not associated with self-care improvements.

**Table 2:** Changes in Diabetes-Related Health Outcomes Associated With a 2-Year Diabetes Self-Management Support Intervention and 1-Year Follow-Up Period for 52 African American Adults With Type 2 Diabetes, Ypsilanti, Michigan, 2006-2009

Variable	2-Year Lifelong Management Intervention				1-Year Follow-Up			
	n	Pre	Post^a^	Post-Pre ∆_1_	n	Pre^a^	Post	Post-Pre ∆_2_
**Clinical indices, mean**								
HbA1c, %	51	7.9 (1.7)	8.0 (2.2)	0.09 (2.2)	51	8.0 (2.2)	7.1 (1.4)	−0.93 (1.6)^b^
Weight, lb	52	206.4 (45.6)	201.6 (39.3)	−4.73 (24.5)	52	201.6 (39.3)	204.8 (43.5)	3.2 (19.0)
Body mass index, kg/m^2^	52	34.7 (7.3)	33.9 (6.2)	−0.80 (4.3)	52	33.9 (6.2)	34.4 (6.5)	0.50 (2.9)
Systolic blood pressure, mm Hg	52	137.9 (18.2)	135.9 (19.6)	−2.08 (19.8)	50	135.7 (19.9)	137.2 (16.3)	1.50 (15.8)
Diastolic blood pressure, mm Hg	52	74.9 (10.3)	74.5 (10.2)	−0.40 (11.9)	50	74.8 (9.9)	78.7 (10.4)	3.86 (11.9)^c^
Serum cholesterol, mg/dL	52	153.1 (34.0)	158.6 (50.3)	5.52 (47.7)	49	156.9 (46.9)	130.5 (44.6)	−26.4 (47.1)^b^
HDL cholesterol, mg/dL	52	52.8 (12.9)	50.7 (14.9)	−2.09 (10.6)	49	49.9 (15.0)	43.4 (16.8)	−6.6 (14.1)^d^
LDL cholesterol, mg/dL	52	96.2 (31.5)	92.1 (40.7)	−4.10 (38.2)	49	90.9 (36.2)	75.6 (31.6)	−15.3 (31.1)^b^
**Self**-**care behaviors^e^**								
Following a healthy diet	52	4.1 (2.2)	4.7 (2.0)	0.64 (2.1)^c^	52	4.7 (2.0)	4.6 (2.2)	−0.15 (2.3)
Spacing carbohydrates	52	3.3 (2.6)	4.1 (2.4)	0.81 (2.0)^d^	52	4.1 (2.4)	3.6 (2.3)	−0.50 (2.6)
Exercising	52	2.2 (2.2)	2.5 (2.3)	0.33 (2.7)	52	2.5 (2.3)	2.0 (2.4)	−0.46 (2.5)
Monitoring blood glucose	52	5.3 (2.4)	5.2 (2.6)	−0.15 (2.2)	52	5.2 (2.6)	5.1 (2.5)	−0.10 (1.9)
Inspecting feet	52	5.5 (2.2)	5.6 (2.3)	0.08 (2.1)	52	5.6 (2.3)	5.5 (2.3)	−0.06 (2.6)
Taking medication	41	6.3 (2.0)	6.1 (2.1)	−0.24 (2.3)	41	6.1 (2.1)	5.9 (2.4)	−0.15 (2.4)
Using insulin	25	3.7 (3.4)	5.0 (3.1)	1.32 (3.2)^c^	21	6.0 (2.4)	6.1 (2.1)	0.14 (1.5)
**Psychosocial indices**								
Quality of life^f^	52	30.2 (15.7)	26.7 (13.8)	−3.52 (10.8)^c^	52	26.7 (13.8)	26.8 (11.6)	−0.13 (8.2)
Empowerment^g^	52	4.0 (0.8)	4.1 (0.9)	0.08 (0.89)	52	4.1 (0.9)	4.0 (0.8)	−0.07 (0.63)

Abbreviations: HbA1c, hemoglobin A1c; HDL, high-density lipoprotein; LDL, low-density lipoprotein.

^a^ Post 2-year intervention and pre 1-year follow-up means may differ because post-pre differences are based on complete pairs.

^b^ *P* < .001.

^c^ *P* < .05.

^d^ *P* < .01.

^e^ Response options range from 0 to 7 days.

^f^ Response options range from 17 to 102 (ranging here from 17 to 78); higher scores indicate poorer diabetes-specific quality of life.

^g^ Responses scored on a 5-point Likert scale (1 = strongly disagree, 5 = strongly agree). Due to a technical error, 1 of the items on the original 8-item scale was dropped. A recalculation of the Cronbach α indicated the reliability for the 7 items was excellent, ranging from .85 to .90, and for the 6 was .86.

### 1-Year follow-up period

Table 2 also presents participants’ clinical, self-care, and psychosocial measures before and immediately following the 1-year follow-up period (ie, phase 3 of the study). Significant improvements were found for HbA1c (*P* < .001), serum cholesterol (*P* < .001), and LDL cholesterol (*P* = .001). DBP was significantly higher (*P* = .03) and HDL cholesterol was significantly lower (*P* = .002). No significant changes were found for weight, BMI, or SBP. No changes were found for self-care behavior, diabetes-specific quality of life, or diabetes empowerment.

## Discussion

One year following an empowerment-based DSMS intervention designed for African Americans, participants sustained the postintervention self-care and quality-of-life improvements and demonstrated further improvements in metabolic and cardiovascular outcomes. Most notable among our findings was the significant improvement in glycemic control associated with the 1-year follow-up period (8.0% vs 7.1%). This −0.93% change in HbA1c is comparable to that achieved from oral agents (23). Consistent with our results, other studies have found significant reductions in glycemic control to emerge at long-term follow-up (18 months and beyond) rather than immediately postintervention (9,24,25). For example, in a study of a diabetes education group visit intervention, HbA1c was significantly lower at 2-year follow-up but not at 1-year postintervention (9). Findings from these studies provide some support for a delayed intervention effect.

That clinical outcomes such as glycemic and lipid control would improve 1-year following intervention withdrawal rather than immediately following the 2-year DSMS intervention is curious. There was no significant change in the percentage of participants who were using insulin and taking cholesterol medication during this time. Although these improvements may be attributed to treatment intensification, it is unlikely that this level of intensification occurred only during the final year of the study. Another viable explanation could be that improvements in lifestyle and behavioral changes achieved in the 2-year DSMS intervention (eg, making good dietary choices, spacing out carbohydrates, using insulin as prescribed) require more time to produce clinical benefits. However, after further statistical examination, we found no relationship between these behavioral and clinical improvements. Future studies should investigate other factors that could contribute to a delayed intervention effect.

Although DBP (74.8 mm Hg vs 78.7 mm Hg) and HDL cholesterol (49.9 mg/dL vs 43.4 mg/dL) appeared to worsen at 1-year follow-up, a mean DBP of 78.7 mm Hg still falls within the recommended target range of at or below 80 mm Hg. Similarly, with regard to cardiovascular disease risk, the ratio of total cholesterol to HDL cholesterol is more clinically meaningful than HDL cholesterol alone. At 1-year follow-up, the mean ratio of total cholesterol to HDL cholesterol was 3.14, which is within the target range (≤4).

Some studies evaluating DSME interventions have incorporated reinforcement during or immediately following the intervention with the objective of promoting continued health improvements. Although some research has found reinforcement to be a critical component for sustained behavior change (12), other research has not produced compelling evidence for its value (14). An example favoring reinforcement is a 4-year study of an intensive, structured hospital-based diabetes education program with a built-in yearly “booster” education session conducted in a group-based setting, where previous self-management topics were discussed and “up-to-date” diabetes care information was presented; in this study, more health-related improvements were evidenced in the intervention group compared with the control group (12). Alternatively, another study used a reinforcement component for a 6-week Internet-based diabetes self-management intervention via a discussion board (over 18 months) where participants could post messages asynchronously, with the goal of fostering reciprocal peer support; in this study, there were almost no intervention-control differences (14). Perhaps the critical question is not whether a reinforcement mechanism is present or absent but rather what type of reinforcement mechanism is employed and how frequently and how long the reinforcement mechanism is used.

Rather than incorporating a reinforcement component into a larger DSME intervention, our DSMS intervention could be conceptualized as a participant-driven reinforcement program in and of itself. Specifically, we invited participants to access support (ie, weekly sessions) as frequently as they needed or as was feasible, given their schedule and life circumstances. In other words, participants had complete control over the frequency and extent of support they sought. Essentially, participants functioned as active agents of their self-management and lifestyle change, and the weekly DSMS sessions served as a resource they could use in their self-management efforts. Considering this interpretation, participants may not have been adversely affected when the intervention was withdrawn. Instead, they were already directing their own self-management practices and decisions and could continue to do so even when the resource was no longer available. Clearly, a goal of DSMS interventions is to foster this type of self-sufficiency and self-efficacy.

Although our attrition rate fell within the expected range, having 33% of participants drop out of the study was not ideal, and we did not follow up with participants regarding reasons for study discontinuation. Subsequent investigations should include a formal protocol to inquire about dropout. This study had other limitations. Because of restricted funding, we were not able to conduct a randomized controlled trial. Without a rigorous methodological design, making definitive conclusions about the efficacy of this intervention was not possible. However, considering the extended length of the intervention and subsequent follow-up period, it is unlikely that improvements could be attributed to factors such as the initial enthusiasm at the start of a study or the attention one receives simply by enrolling in a study. Similarly, any changes associated with secular trends would have more likely reflected health deterioration rather than health improvement, particularly for this sample of older participants. Regardless, future investigations should use a randomized controlled trial or comparative effectiveness design to answer these questions.

Effective diabetes self-management requires initial DSME followed by ongoing DSMS (16). Given the overwhelming evidence for the short-term effect of diabetes self-management interventions, greater attention is needed for developing, implementing, and evaluating interventions that yield positive outcomes that are sustained throughout life. Interest in examining peer support models as an effective and viable approach to long-term DSMS is growing, and understanding which aspects or underlying mechanisms of self-management interventions lead to long-lasting diabetes-related health benefits is needed.
